# A bradykinin-potentiating peptide (BPP-10c) from Bothrops jararaca induces changes in seminiferous tubules

**DOI:** 10.1186/1678-9199-19-28

**Published:** 2013-11-06

**Authors:** Joyce M Gilio, Fernanda CV Portaro, Maria I Borella, Claudiana Lameu, Antonio CM Camargo, Carlos Alberto-Silva

**Affiliations:** 1 Center for Applied Toxinology (CAT-CEPID), Butantan Institute, São Paulo, São Paulo State, Brazil; 2 Laboratory of Immunochemistry, Butantan Institute, São Paulo, São Paulo State, Brazil; 3 Department of Cell and Developmental Biology, Laboratory of Fish Endocrinology, Institute of Biomedical Sciences, University of São Paulo (USP), São Paulo, São Paulo State, Brazil; 4 Department of Biochemistry, Chemistry Institute, University of São Paulo (USP), São Paulo, São Paulo State, Brazil; 5 Natural and Human Sciences Center (CCNH), Federal University of ABC (UFABC), Santo André, São Paulo State, Brazil

**Keywords:** Bradykinin-potentiating peptide, Snake venom, Angiotensin-converting enzyme, Testis, Seminiferous epithelium

## Abstract

**Background:**

The testis-specific isoform of angiotensin-converting enzyme (tACE) is exclusively expressed in germ cells during spermatogenesis. Although the exact role of tACE in male fertility is unknown, it clearly plays a critical function in spermatogenesis. The dipeptidase domain of tACE is identical to the C-terminal catalytic domain of somatic ACE (sACE). Bradykinin potentiating peptides (BPPs) from snake venoms are the first natural sACE inhibitors described and their structure–activity relationship studies were the basis for the development of antihypertensive drugs such as captopril. In recent years, it has been showed that a number of BPPs – including BPP-10c – are able to distinguish between the N- and C-active sites of sACE, what is not applicable to captopril. Considering the similarity between tACE and sACE (and since BPPs are able to distinguish between the two active sites of sACE), the effects of the BPP-10c and captopril on the structure and function of the seminiferous epithelium were characterized in the present study. BPP-10c and captopril were administered in male Swiss mice by intraperitoneal injection (4.7 μmol/kg for 15 days) and histological sections of testes were analyzed. Classification of seminiferous tubules and stage analysis were carried out for quantitative evaluation of germ cells of the seminiferous epithelium. The blood-testis barrier (BTB) permeability and distribution of claudin-1 in the seminiferous epithelium were analyzed by hypertonic fixative method and immunohistochemical analyses of testes, respectively.

**Results:**

The morphology of seminiferous tubules from animals treated with BPP-10c showed an intense disruption of the epithelium, presence of atypical multinucleated cells in the lumen and degenerated germ cells in the adluminal compartment. BPP-10c led to an increase in the number of round spermatids and total support capacity of Sertoli cell in stages I, V, VII/VIII of the seminiferous epithelium cycle, without affecting BTB permeability and the distribution of claudin-1 in the seminiferous epithelium. Interestingly, no morphological or morphometric alterations were observed in animals treated with captopril.

**Conclusions:**

The major finding of the present study was that BPP-10c, and not captopril, modifies spermatogenesis by causing hyperplasia of round spermatids in stages I, V, and VII/VIII of the spermatogenic cycle.

## Background

Spermatogenesis takes place in the seminiferous epithelium of the mammalian testis. The germ cells residing in the basal compartment must traverse the blood-testis barrier (BTB) and enter the adluminal compartment for further development into round, elongated spermatids [1]. The inter-Sertoli tight junctions (TJ) constitute the BTB that protects the seminiferous epithelium from invasion by molecules or cells that may disturb the process of spermatogenesis. At the same time, this permeability barrier needs to be temporarily removed at particular stages of spermatogenesis for the movement of germ cells across the seminiferous epithelium [2]. TJ is a multimolecular membrane that comprises integral membrane proteins, including occludin and claudin family proteins [2,3].

Testis-specific isoform of angiotensin-converting enzyme (tACE) is exclusively expressed in maturing germ cells and spermatozoa, but not in Sertoli cells, Leydig cells or any other somatic cell in male adults, suggesting that it is related to the spermiogenesis process [4-7]. Experimental evidence using tACE knockout models (−/−) indicates that this enzyme is directly associated with male fertility, but its exact role remains unknown [5,8-11]. It has been reported that tACE is able to release the extracellular portion of glycosylphosphatidylinositol (GPI)-anchored proteins, and it is directly and specifically implicated in egg fertilization by the sperm, independent of its peptidase activity [12,13].

Somatic angiotensin I-converting enzyme (sACE) is a well-characterized zinc dipeptidyl carboxypeptidase that plays a pivotal role in the regulation of blood pressure by converting angiotensin I into angiotensin II and by inactivating bradykinin [14]. sACE has two highly homologous active sites, one at the C-domain and another at the N-domain, each of which is catalytically active and functionally independent [15]. tACE is distinguishable from sACE because it has only the active site of the C-domain, preceded by an additional N-terminal sequence [16].

Bradykinin potentiating peptides (BPPs) from *Bothrops jararaca* snakes were the first natural sACE inhibitors described. Studies of their structure–activity relationships were the basis for the development of antihypertensive drugs, such as captopril [17]. Typically, BPPs contain 5 to 13 amino acid residues with a pyroglutamyl residue (<E) at the N-terminus and a proline residue at the C-terminus. BPPs longer than seven amino acids share similar features, including a high content of proline residues and the tripeptide sequence Ile–Pro–Pro at the C-terminus [18].

We found that BPP-10c (<ENWPHPQIPP) is able to distinguish between the two domains of sACE and displays distinct hypotensive effects on rats [19,20]. In addition, among other BPPs from snake venom, BPP-10c is the most selective inhibitor for the active site at the C-domain of sACE (K_i(app)_ = 0.5 nM) [19]. Captopril, for instance, is 2.8-orders of magnitude less effective than BPP-10c as an inhibitor of the C-site of sACE [21]. In recent years, we have supported the hypothesis that diverse biological functions for each BPP could be mediated by different interactions with alternative targets, including that BPP-10c is internalized by HUVEC, HEK293 and C6 cells [22-25]. These results are not surprising, considering that BPP-10c is a proline-rich peptide, a feature that endows this molecule with the properties of cell-penetrating peptides and resistance to proteolysis.

Considering the structural similarity between the C-domain of sACE and tACE, it was observed that tACE male knockout mice were severely hypofertile, tACE was exclusively expressed in maturing germ cells, BPP-10c had selectivity for the active site at the C-domain of sACE and it could be internalized by different cells, and ACE inhibitors could affect the function of the seminiferous epithelium, particularly spermiogenesis [5,7,9,18,22,24,25]. Although the *in vitro* nanomolar range inhibition of human tACE by BPP-5a (<EKWAP) and BPP-9a (<EWPRPQIPP) has been reported, there are no reports on the possible effects of BPPs in the structure and function of the seminiferous epithelium [26]. Thus, the aim of the current study was to compare the effect of BPP-10c and captopril on spermatogenesis in male mice in order to evaluate the morphological and morphometric parameters, distribution of claudin-1 and analysis of BTB permeability in the seminiferous epithelium.

## Methods

### Animals

Male Swiss mice (weighting 30 to 35 g) were bred at the Butantan Institute (São Paulo, Brazil). Animals were housed at a temperature of 22°C, had access to water and food *ad libitum*, and were subjected to a light–dark cycle (12 hours each). The experimental protocols were performed in accordance with the guidelines of the Butantan Institute for the humane use of laboratory animals and were approved by local authorities (protocol number 369/07).

### Reagents

All chemicals were of analytical reagent grade, purchased from Calbiochem-Novabiochem Corp. (USA), Merck (USA) and Sigma–Aldrich Corp. (USA) for peptide synthesis; captopril and bradykinin were purchased from Sigma Chemical Co (USA).

### Peptide synthesis

BPP-10c (<ENWPHPQIPP) was synthesized using automated solid-phase synthesis via Fmoc (9-fluorenylmethyloxycarbonyl) strategy [23]. The final deprotected peptide was purified by semi-preparative HPLC using an Econosil C-18 column (10 μm, 22.5 mm × 250 mm) and a two-solvent system: (A) TFA/H_2_O (1:1000) and (B) TFA/ACN/H_2_O (1:900:100). The column was eluted at a flow rate of 5 mL/minute over 20 minutes with a 10 to 50% gradient of solvent B, and the effluent was detected with an SPD-10AV Shimadzu UV–vis detector, monitored by absorbance at 220 nm. The molecular weight and purity of synthetic peptide were checked via MALDI-TOF mass spectrometry using an Ettan MALDI-TOF/Pro system (Amersham Biosciences, Sweden) and cinnamic acid as a matrix. The peptide concentration was determined by amino acid analysis after acid hydrolysis in vacuum-sealed tubes at 110°C for 22 hours with HCl 6 N containing 1% phenol. Samples were subjected to amino acid analysis using a pico Tag station.

### Treatment of animals with BPP-10c and captopril

Male adult mice (30–35 g) were assigned to groups (five animals per group) and treated for 15 days (once a day) by intraperitoneal injection with 4.7 μmol/kg/day of BPP-10c or captopril, diluted in 100 μL of 0.91% w/v aqueous sodium chloride solution. The control group consisted of treatment with vehicle only. The dose of BPP-10c used in the experiments was in agreement with Silva *et al*. [23]. Mice were killed by CO_2_ asphyxiation after treatment and testes were collected for morphological, morphometric and immunohistochemical analyses of seminiferous epithelium. BTB permeability studies were carried out in mice treated with BPP-10c, captopril (the same dose, 4.7 μmol/kg/day), lipopolysaccharide (LPS, 166 μmol/kg/day – positive control) or vehicle (0.91% w/v aqueous sodium chloride solution – negative control) for 15 days. All treatments and experiments were performed in duplicate or triplicate.

### Morphological and morphometric analyses

The testes of mice treated with BPP-10c, captopril or vehicle were immediately immersed and fixed in Bouin’s solution for 24 hours. The samples were dehydrated in ethanol, and embedded in Paraplast® (Sigma Chemical Co., USA) and sectioned at 4 μm thickness. Histological sections were stained with periodic acid-Schiff’s (PAS) with Harris hematoxylin counterstaining (for morphometric analysis), or Mallory's trichrome stain (for morphological analysis). Images were taken using a Pixera camera (Pixera Corporation, USA) mounted on a Zeiss Axioskop 2 photomicroscope and captured with a Intel Pentium® computer using Adobe Photoshop 7.0.1 (Adobe Systems, USA).

The stages of the seminiferous epithelium cycle were characterized based on the development of the acrosomic system and morphology of the developing spermatid nucleus [27]. Four spermatogenic stages (I, V, VII/VIII, and XII), representing beginning, middle and end of seminiferous epithelium cycle were chosen for quantitative evaluation [28]. Four round or nearly-round seminiferous tubule cross-sections per animal were randomly selected for each spermatogenic stage and the following parameters were measured: epithelium height, tubule diameter and lumen diameter using NIH image software (developed at the U.S. National Institutes of Health and available at http://rsb.info.nih.gov/nih-image). The germ cell nuclei (type A spermatogonia; type B spermatogonia; preleptotene spermatocyte; zygotene spermatocyte; meiotic figures; secondary spermatocyte; pachytene spermatocyte; round spermatid) and Sertoli cell nucleoli present at stages I, V, VII/VIII, and XII of the seminiferous epithelium cycle were counted using Adobe Photoshop 7.0.1. Total support capacity of each Sertoli cell was obtained by the ratios of total number of germ cells to total number Sertoli cell nucleoli for each stage.

### Distribution of claudin-1 by immunohistochemistry

Testis sections from mice treated with BPP-10c, captopril or vehicle were processed according to the Streptavidin-Biotin-peroxidase Complex (SBC) protocol. After deparaffinization and dehydration, the sections were pretreated with 0.03% H_2_O_2_ for 30 minutes, at room temperature, to block endogenous peroxidase activity. Samples were then washed in phosphate-buffered saline pH 7.4 (PBS), two times for 5 minutes each, and immersed in a solution containing 5% fat-free dry milk (Molico, Nestlé®) in PBS for 15 minutes to block non-specific binding sites.

Sections were incubated overnight at 4°C with anti-rabbit claudin-1 antiserum (MH25- Zymed/Invitrogen®, lot 50393527, cat. no. 71–7800) diluted (1:250) in 0.05 M Tris–HCl with 1% bovine serum albumin (BSA). They were washed in PBS three times for 5 minutes and incubated with the diluted biotinylated anti-rabbit IgG for 30 minutes, then washed in PBS three times for 5 minutes and incubated for 30 minutes with Streptavidin-biotin-peroxidase complex. Immunoreactive sites were revealed using a buffered solution of 3,3’-diaminobenzidine–tetrahydrochloride (DAB) (Dako cytomation®, USA). The sections were dehydrated, mounted and analyzed in a Zeiss Axioskop 2 photomicroscope and the images were captured by Pixera (Pixera Corporation, USA). As negative control, normal rabbit IgG (Vector Laboratories) was used instead of the first antibody in every experiment. Hematoxylin was used for counterstaining. We also performed immunoblot analysis of mouse testis lysate to assess the specificity of anti-rabbit claudin-1 antibody.

### Analysis of BTB by hypertonic fixative method

Mice treated with BPP-10c, captopril, LPS or vehicle were perfused with a hypertonic fixative of 5% glutaraldehyde, 0.05 M sodium cacodylate, and 10% dextrose [29]. Testes were removed and fixed by immersion in hypertonic fixative for 2 hours at 4°C. The testes were washed in 0.05 M sodium cacodylate buffer for 10 minutes, cut in half transversely, post-fixed in 1% osmium tetroxide, and embedded in Paraplast® following standard procedures. Half-μm-thick sections were stained with hematoxylin and eosin.

### Statistical analysis

All statistical evaluations were performed either by one-way analysis of variance (ANOVA) followed by the Bonferroni range test or by Student’s *t*-test (GraphPad Prism 4.0, GraphPad Software, Incorporation). The criteria for statistical significance were set at *p <* 0.05.

## Results

### Effect of BPP-10c on the seminiferous epithelium in male adult mice

**Figure 1:**
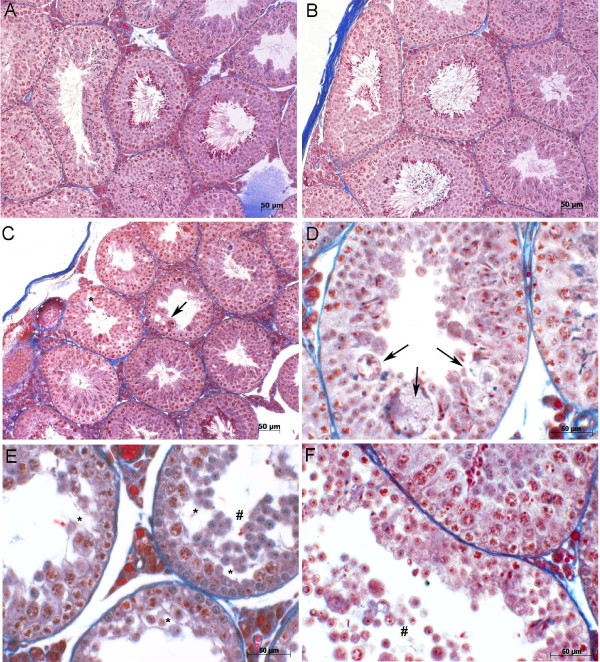
**Morphological analysis of the seminiferous epithelium of male adult mice treated with BPP-10c and captopril.** Photomicrographs of the seminiferous tubules of male adult mice treated with vehicle **(A)**, captopril **(B)** and BPP-10c **(C, D, E, F)** using Mallory’s trichrome stain. The seminiferous tubule morphological data obtained from the animals treated with BPP-10c indicated the presence of atypical cells in the lumen (arrow), disruption of the epithelium (*), and a loss of elongated spermatids (#). No alteration was observed in the seminiferous epithelium from mice treated with captopril or vehicle.

The seminiferous tubules observed in animals treated with vehicle (Figure 1 – A) and captopril (Figure 1 – B) displayed normal testicular tissue with typical seminiferous epithelium after 15 days of treatment. In contrast, testes of animals treated with BPP-10c (Figure 1 – C to F) presented atypical multinucleated cells in the lumen, degenerated germ cells in the adluminal compartment, disruption of the epithelium, and loss of elongated spermatids in the tubules. No alterations in the number of spermatogonia, preleptotene spermatocytes, zygotene spermatocytes, pachytene spermatocytes, or Sertoli cells were detected after treatment with BPP-10c, captopril or vehicle (Table 1). However, the treatment with BPP-10c led to an increase in the number of round spermatids in stages I, V, VII/VIII (Table 1).

**Table 1:** Quantitative analysis of germinal cells in male adult mouse seminiferous epithelium treated with vehicle (C), captopril (CAP) or BPP-10c (10c)

**Germinal cells**	**Stages**											
	**I**			**V**			**VII/VIII**			**XII**		
	**C**	**10c**	**CAP**	**C**	**10c**	**CAP**	**C**	**10c**	**CAP**	**C**	**10c**	**CAP**
**SC**	4.0 ± 0.8	4.7 ± 0.5	5.0 ± 0.8	4.5 ± 0.5	4.2 ± 0.9	5.0 ± 0.9	5.2 ± 0.5	4.7 ± 0.9	5.1 ± 0.4	5.2 ± 0.5	5.5 ± 0.5	5.8 ± 0.9
**SPG(A)**	3.5 ± 0.5	4.0 ± 0.5	4.6 ± 0.9									
**SPG(B)**				5.2 ± 0.5	4.5 ± 0.5	5.5 ± 0.5						
**SP(Pl)**							13.2 ± 0.5	13.0 ± 0.8	12.7 ± 1.2			
**SP(Z)**										12.2 ± 0.5	11.0 ± 0.8	12.4 ± 1.5
**MF**										9.7 ± 0.5	9.0 ± 0.5	8.9 ± 0.8
**SS**										6.2 ± 0.5	5.2 ± 0.9	5.1 ± 0.9
**SP(P)**	10.5 ± 0.5	10.0 ± 0.8	10.9 ± 1.0	12.0 ± 0.8	12.2 ± 0.8	13.0 ± 1.2	19.2 ± 0.5	19.0 ± 0.8	20.1 ± 1.5			
**RP**	14.0 ± 0.8	27.0 ± 0.9*	15.0 ± 1.8	14.7 ± 0.5	19.0 ± 0.8*	15.7 ± 0.9	15.0 ± 0.8	30.0 ± 0.8*	16.2 ± 0.8			

C: control; 10c: BPP-10c; CAP: captopril; SC: Sertoli cell; SPG(A): type A spermatogonia; SPG(B): type B spermatogonia; SP (Pl, Z, P): spermatocyte (preleptotene zygotene, pachytene); MF: meiotic figures; SS: secondary spermatocyte; RP: round spermatid.

Values are the means ± SEM. Significant difference from the C and CAP values *p* < 0.01 (*).

**Figure 2:**
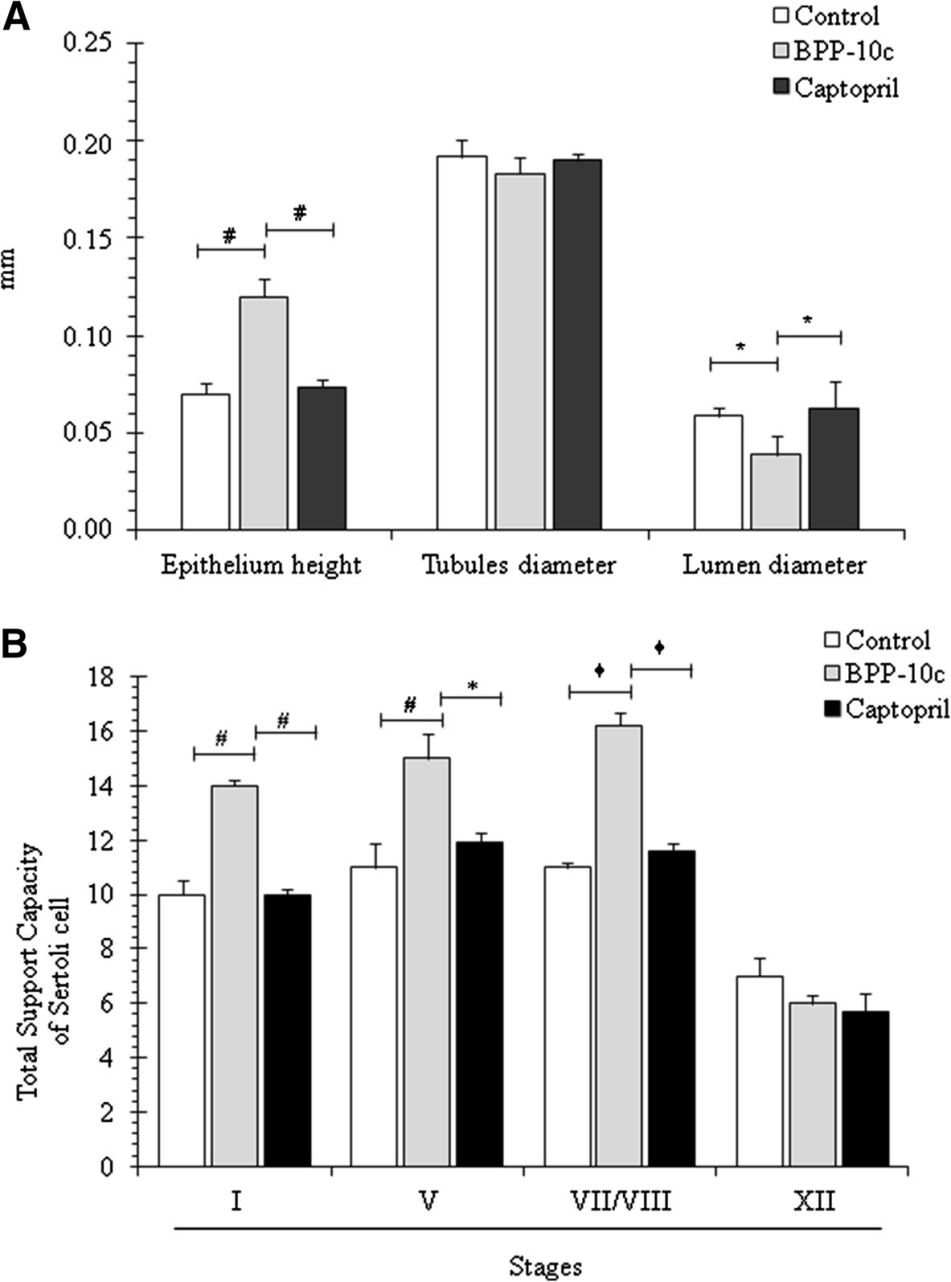
**Morphometric analysis of the seminiferous epithelium of male adult mice treated with BPP-10c or captopril. (A)** Morphometric aspects of the seminiferous tubules of control and treated animals (epithelium height, tubule diameter and lumen diameter). **(B)** Total support capacity of Sertoli cell increased during stages I, V, VII/VIII and XII of the seminiferous epithelium cycle when animals were treated with BPP-10c. Data are presented as mean ± SEM, and the criteria for statistical significance were set at *p* < 0.05.

Epithelium height increased and lumen diameter decreased in the testes of the animals treated with BPP-10c, but no alteration was detected in the tubule diameter when compared with vehicle (Figure 2 – A). Furthermore, BPP-10c increased the total support capacity of Sertoli cell in stages I, V, VII/VIII of the seminiferous epithelium cycle, but not in stage XII (Figure 2 – B). Interestingly, no significant differences were showed in morphometrical parameters analyzed in the testis of the animals treated with captopril when compared with vehicle or BPP-10c (Figure 2 – A and B).

### Immunohistochemical localization of claudin-1 in the seminiferous epithelium after BPP-10c treatment

**Figure 3:**
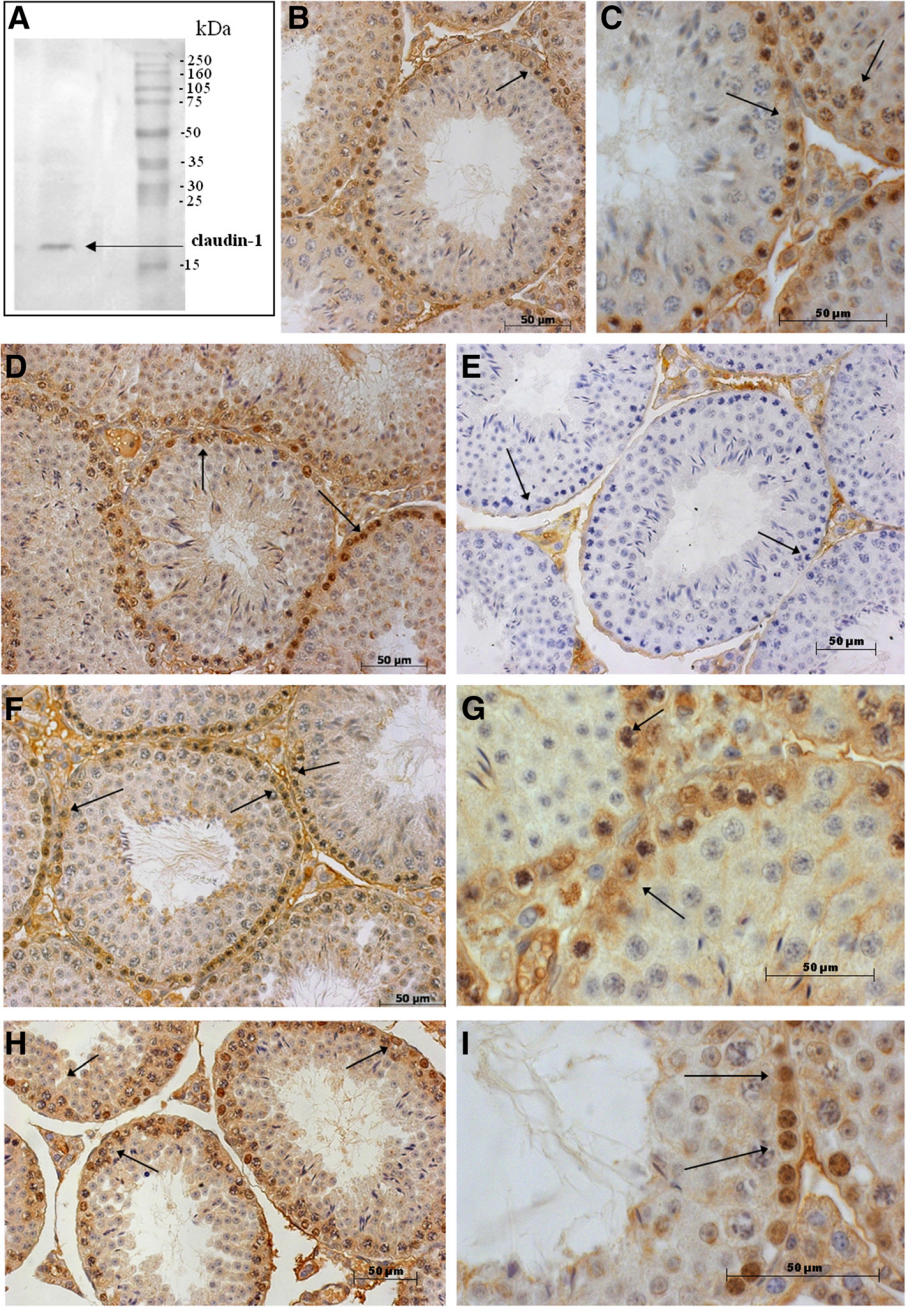
**Effects of BPP-10c and captopril on the distribution of claudin-1 in the seminiferous epithelium of adult mouse testis. (A)** Immunoblot analysis of mouse testis lysate using an antibody to claudin-1. **(B, C, D)** Immunohistochemical staining of mouse testis transverse cross-sections treated with vehicle. **(E)** Non-specific staining was detected only in the basal and adluminal compartments of seminiferous epithelium of control sections – negative control. **(F and G)** Immunostaining of claudin-1 following treatment with captopril or **(H and I)** BPP-10c demonstrated no difference in the distribution of claudin-1 when compared with control. Hematoxylin was used for counterstaining. Scale bar: 50 μm.

Claudin-1 was observed in the mouse testis tissue lysates through immunoblot analysis using anti-claudin-1 antibody, detected as a single band at approximately 20 kDa (Figure 3–A). The localization of immunoreactive claudin-1 in the seminiferous epithelium appeared as reddish-brown precipitate in the basal compartment of normal mouse seminiferous tubules (Figure 3 – B, C and D). Non-specific staining was only detected in the seminiferous epithelium of control sections, illustrating that the immunoreactivity is specific for claudin-1 (Figure 3 – E). Immunoreactive claudin-1 precipitates formed in the basal and adluminal compartments of each tubule in every stage of the germinal epithelium cycle. Moreover, immunoreactive specificity could be observed in the nucleus of premeiotic germ cells, but not in pachytene spermatocytes, secondary spermatocytes, or round spermatids. Likewise, the distribution of claudin-1 following captopril (Figure 3 – F and G) and BPP-10c (Figure 3 – H and I) treatments was the same as that of seminiferous epithelium in untreated mice.

### Analysis of BTB permeability

**Figure 4:**
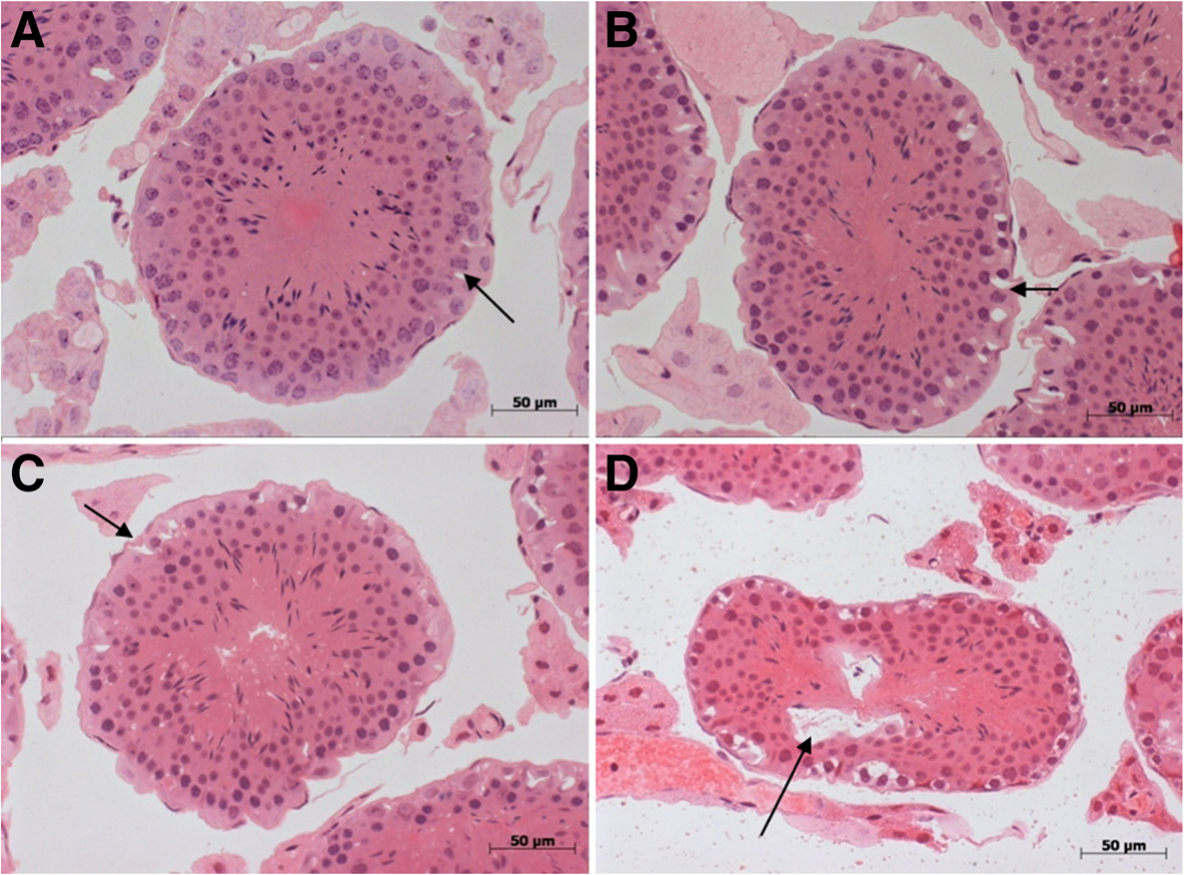
**Maintenance of the blood-testis barrier (BTB) in male adult mice treated with vehicle, captopril or BPP-10c.** Photomicrographs of 0.5-μm thick – plastic-embedded transverse sections of **(A)** vehicle, **(B)** captopril, **(C)** BPP-10c and **(D)** LPS – of seminiferous tubules following fixation with hyperosmotic fixative and staining with hematoxylin and eosin. The adluminal meiotic cells are protected from fixative-induced condensation, maintaining normal morphology and cellular contacts in **A**, **B** and **C** (arrow). The BTB is not maintained in the seminiferous tubules of mice treated with LPS (**D**, see arrow).

The barrier can be visualized in testes fixed with a dextrose-containing hypertonic fixative. The permeability of BTB was examined in testicular sections of mice treated with vehicle, captopril, BPP-10c or LPS. Cells directly exposed to the fixative, such as those on the basal side of the barrier, suffer the osmotic effects of the dextrose and shrink, leaving large intercellular gaps. Because dextrose cannot transverse the BTB, cells on the luminal side are protected from the hypertonicity of the fixative and maintain close apposition with one another. The BTB was maintained in testes of mice treated with vehicle, captopril and BPP-10c (Figure 4 – A, B and C, respectively, see arrows). However, the barrier was not intact following LPS treatment and cells throughout the cross-section of the tubule shrank away from each other (Figure 4 – D, see arrow). LPS is known to compromise testicular function [30].

## Discussion

The major finding of the present study was that BPP-10c, the most potent and selective sACE C-domain inhibitor, modified spermatogenesis in mice without affecting BTB permeability or the distribution of claudin-1, a protein found at the site of the BTB [19]. Interestingly, captopril, which is also an inhibitor of sACE, did not show any effect on spermatogenesis probably due to the inability of this ACE active-site directed inhibitor to cross the BTB. In fact, it has been described that sACE inhibitors (captopril and derivates) did not affect tACE activity *in vivo*, suggesting that these drugs are limited in testicular penetration by the BTB [30,31].

Morphologic investigation of testes in adult mice indicated a clear alteration in the seminiferous epithelium following BPP-10c treatment. Alterations were observed in stages I, V, VII/VIII in round spermatids, while no such alterations were observed following captopril treatment. The effects of BPP-10c treatment also included an increase in the height of the epithelium and a decrease in the diameter of the tubule lumen, as well as an increase in the total support capacity of Sertoli cells, indicating that BPP-10c inhibited spermiogenesis. In fact, tACE is only found in round spermatids in the seminiferous tubules, with levels increasing markedly during further differentiation [7]. The observed effects of BPP-10c could be explained by the interaction with tACE, which causes alterations in its dipeptidase and/or GPI-anchored protein-releasing activities, leading to an inhibition of maturation with an increase in the number of round spermatids.

Claudin-1 expression has been demonstrated in the testis and culture of mouse Sertoli cells, but we have shown in the current study for the first time its distribution in mouse seminiferous epithelium [32]. Other studies have indicated that claudin-1 expression in the epididymis is not exclusively limited to TJ, but appears along the entire interface of adjacent epithelial cells, as well as along the basal plasma membrane [33]. We demonstrated that claudin-1 is found in the basal and adluminal compartments in the seminiferous epithelium of normal mice, suggesting that claudin-1 may have functions other than those involving TJ in the testis and epididymis. Moreover, Gregory *et al*. [34] identified claudin-1 in the nucleus as an intracellular signaling molecule that either diffuses or is actively transported to the nucleus from the site of cell-cell adhesion by mitogen-activated protein kinase kinase 2 (MEK2) in pancreatic cells. MEK2, an isoform of mitogen-activated protein kinases (MAPKs), is found in all premeiotic germ cells and spermatocytes, and is implicated in chromatin condensation during the division of male germ cells [35-37]. In fact, our immunohistochemical studies showed that claudin-1 is expressed in premeiotic germ cells, with a pattern similar to that of MEK2 expression. These results suggest that claudin-1 participates in chromatin condensation of germ cells by signaling through MEK2.

The localization of claudin-1 in the seminiferous epithelium was examined to assess its possible changes in the BTB during the BPP-10c-induced spermatogenesis damage. No alterations were shown in the distribution of claudin-1 in animals treated with BPP-10c, captopril, or vehicle, suggesting that the peptide did not alter BTB integrity. Some chemicals disrupt this barrier and increase its permeability, but we have shown that treatment with BPP-10c did not alter BTB permeability, suggesting that the peptide could cross the BTB and interact with tACE or others targets [30,38,39]. This hypothesis seems to represent a real possibility, since a growing number of peptides, including toxins, have been shown to penetrate cells [25,40,41]. Besides, we demonstrated that BPP-10c was internalized by HEK-293 T and HUVEC cells, but the mechanism is yet unknown and it opens new perspectives to study their internalization by Sertoli cell culture [23,24].

## Conclusion

In summary, this study demonstrated that BPP-10c, a potent selective C-domain inhibitor of ACE from *B. jararaca* venom, inhibits spermiogenesis in mice without affecting the distribution of claudin-1 or the permeability of BTB. Further analyses will contribute to a better understanding of the molecular mechanism underlying the effects of BPPs from snake venom in the testicular physiology, adding new biological features to the whole venom.

### Ethics committee approval

All experimental protocols described in the present study were performed in accordance with the guidelines for use of laboratory animals of Butantan Institute and approved by local authorities (protocol number 369/07).

## Competing interests

The authors declare that there are no competing interests.

## Authors’ contributions

JMG carried out most experiments assisted by other researchers. FCVP, MIB and CL contributed to the design of experiments. ACMC established the conditions for the study. CAS established the conditions for the study, was responsible for drafting the manuscripts, reading it and for the editorial corrections. All authors read and approved the final manuscript.
